# Acute Lyme Disease With Atypical Features due to *Borrelia mayonii*

**DOI:** 10.1093/ofid/ofad524

**Published:** 2023-10-24

**Authors:** Mitchell S McGowan, Thomas M Kalinoski, Shayla E Hesse

**Affiliations:** Des Moines University School of Medicine, Des Moines, Iowa, USA; Department of Internal Medicine, Sanford Health, Bemidji, Minnesota, USA; Department of Infectious Diseases, Sanford Health, Bemidji, Minnesota, USA

To  The  Editor—Recent estimates suggest that >475 000 Americans are diagnosed and treated for Lyme disease yearly [1]. In 2016, a new causative agent of Lyme borreliosis was identified: *Borrelia mayonii*, aptly named after the institutional affiliation of its discoverers [2]. Now, >7 years later, only 7 cases of Lyme disease due to *B mayonii* have been described—including 6 cases contained within the original report of the pathogen [2, 3]. Lest there be any doubt of the ongoing clinical relevance of *B mayonii* in the upper Midwest, we describe a recent case from northern Minnesota. Opportunities for increased recognition of this Lyme disease variant are highlighted.

A previously healthy 51-year-old man presented to the hospital in July with a 2-day history of fever, shaking chills, intense headache, intermittent confusion, diffuse arthralgias, dry cough, generalized weakness, and mild dyspnea. For the past 2 weeks, he had been felling trees and clearing brush on his property in rural Minnesota. He was febrile to 39.1 °C and mildly tachycardic. On examination, patches of confluent macular erythema were appreciated on his torso without accompanying neck stiffness, joint effusion, or focal neurologic deficit. Basic laboratory studies were unremarkable apart from mild lymphocytopenia and elevated inflammatory markers (C-reactive protein, 125.7 mg/L; procalcitonin, 0.75 ng/mL). Lumbar puncture was successful on the second attempt. Cerebrospinal fluid (CSF) analysis signaled a traumatic tap but was otherwise unremarkable (4039 red blood cells/μL, 6 white blood cells/μL; protein, 34 mg/dL; glucose, 83 mg/dL). Computed tomography of the head was negative for intracranial bleed.

Multiplex polymerase chain reaction (PCR) testing for Lyme disease pathogens in the CSF flagged positive for *B mayonii*. Other *Borrelia* species, including *B burgdorferi*, *garinii*, and *afzelii*, were not detected. Lyme disease serology was negative, supporting a diagnosis of acute infection. A broad microbiologic workup excluded coinfection with *Anaplasma*, *Ehrlichia*, *Babesia*, *Rickettsia*, *Blastomyces*, *Histoplasma*, or West Nile virus.

Despite the positive CSF PCR finding, we deemed central nervous system infiltration of *B mayonii* to be improbable. Unlike *B burgdorferi*, *B mayonii* achieves high levels of spirochetemia, thereby rendering its molecular detection from blood-contaminated CSF difficult to interpret. A Lyme disease antibody index from paired serum and CSF samples was expected to be inconsequential given the acuity of the infection and thus was not assessed. Regardless, the patient was treated for neuroborreliosis with 5 days of intravenous ceftriaxone, followed by 16 days of oral doxycycline. He made a full recovery. Lyme seroconversion, including IgM immunoblot confirmation, was verified at clinic follow-up 3 weeks postsymptom onset.

Several aspects of our patient's presentation were atypical for acute borreliosis due to *B burgdorferi*, including patchy macular erythema, high fever, and encephalopathy. Notably, each of these signs/symptoms was present in at least 50% of the *B mayonii*–infected patients described in the original report, providing potential clues for diagnosis [2]. For patients who are acutely ill and have at least 2 of the aforementioned clinical features plus risk factors for Midwestern tick exposure, we propose that the diagnostic workup routinely include molecular testing for *B mayonii* in the blood and/or CSF. Based on the clinical data accumulated to date, high spirochetemia associated with acute *B mayonii* infection confers sufficient PCR sensitivity to justify this approach. The upshot, besides facilitating a clearer epidemiologic profile of a relatively new pathogen, includes the opportunity to diagnose acute Lyme disease in the absence of classical erythema migrans and prior to Lyme seroconversion.

## Notes

***Patient consent statement*.** Written consent was obtained from the patient for publication of this case report.
